# Total coliforms as an indicator of human enterovirus presence in surface water across Tianjin city, China

**DOI:** 10.1186/s12879-018-3438-5

**Published:** 2018-11-01

**Authors:** Jing Miao, Xuan Guo, Weili Liu, Dong Yang, Zhiqiang Shen, Zhigang Qiu, Xiang Chen, Kunming Zhang, Hui Hu, Jing Yin, Zhongwei Yang, Junwen Li, Min Jin

**Affiliations:** 1 Tianjin Institute of Environmental & Operational Medcine, Key Laboratory of Risk Assessment and Control for Environment & Food Safety, Tianjin, 300050 China; 2 Research Institution of Chemical Defense, Beijing, 102205 China

**Keywords:** Surface water, Human enteric viruses, Total coliforms, Viral indicator

## Abstract

**Background:**

Enteric viruses in surface water pose considerable risk to morbidity in populations living around water catchments and promote outbreaks of waterborne diseases. However, due to poor understanding of the correlation between water quality and the presence of human enteric viruses, the failure to assess viral contamination through alternative viral indicators makes it difficult to control disease transmission.

**Methods:**

We investigated the occurrence of Enteroviruses (EnVs), Rotaviruses (HRVs), Astroviruses (AstVs), Noroviruses GII (HuNoVs GII) and Adenoviruses (HAdVs) from Jinhe River over 4 years and analyzed their correlation with physicochemical and bacterial parameters in water samples.

**Results:**

The findings showed that all target viruses were detected in water at frequencies of 91.7% for HAdVs, 81.3% for HuNoVs GII, 79.2% for EnVs and AstVs, and 70.8% for HRVs. These viruses had a seasonal pattern, which showed that EnVs were abundant in summer but rare in winter, while HAdVs, HRVs, AstVs, and HuNoVs GII exhibited opposite seasonal trends. Pearson correlation analysis showed that total coliforms (TC) was significantly positively correlated with EnVs concentrations while no consistent significant correlations were observed between bacterial indices and viruses that precipitate acute gastroenteritis.

**Conclusions:**

Taken together, the findings provide insights into alternative viral indicators, suggesting that TC is a potentially promising candidate for assessment of EnVs contamination. However, it failed to predict the presence of HAdVs, HRVs, AstVs, and HuNoVs GΙΙ in surface water across the city of Tianjin.

**Electronic supplementary material:**

The online version of this article (10.1186/s12879-018-3438-5) contains supplementary material, which is available to authorized users.

## Background

Human enteric viruses are a diverse group of organisms including Enteroviruses (EnVs), Rotaviruses (HRVs), Astroviruses (AstVs), Noroviruses GII (HuNoVs GII), and Adenoviruses (HAdVs). These viruses are transmitted by the fecal-oral route, resulting in a wide range of waterborne diseases, such as gastroenteritis and hepatitis [1]. According to the World Health Organization (WHO), human enteric viruses infect billions of people every year and the resultant diarrheal disease accounts for the second leading cause of death in children under 5 years globally [2]. They pose an especially high risk of mortality in young children, immune-compromised patients, and the elderly [3].

The high infection rates and rapid transmission between humans contribute to the significant morbidity associated with enteric viruses [4]. The absence of specific vaccines for human enteric viruses (except HRVs) further enhances the risk of infection [5]. More importantly, exposure to enteric virus-contaminated environments and/or drinking water, are generally thought to be responsible for a large proportion of outbreaks of waterborne diseases [6]. In recent years, there have been significant efforts to investigate human enteric viruses in water sources. Almost all types of human enteric viruses, including HAdVs, HRVs, AstVs, EnVs, and HuNoVs GII, have been found in surface water [7, 8], sewage [9], recreational water [10], raw water sources [11], and seawater [12].

The low viral removal efficiency of wastewater treatment and strong intrinsic resistance to the water disinfection processes [13], may aid viral survival in water [14], resulting in contamination of viruses in water. However, there are no ideal indicators that are typically correlated with or specific enough to predict the presence of human enteric viruses. This leads to the failure in assessing the occurrence of enteric viruses in surface water, which may be another important consideration for why viral contamination in water has been overlooked [15]. This is the case even where bacterial indicators are currently used in most countries around the world for assessment of fecal or pathogen contamination in surface water [16]. In such settings, it is essential to explore a sensitive indicator that sufficiently expedites an early warning system relating to the occurrence of enteric viruses, which enables corrective action to be applied in a timely manner in regions with enteric virus-contaminated waters.

Here, we investigated the prevalence of five enteric viruses, including HAdVs, HRVs, AstVs, EnVs, and HuNoVs GII, in the Jinhe River of Tianjin city over 4 years. By analyzing the correlations between viral prevalence in the river and bacterial indices e.g., heterotrophic plate counts (HPC), total coliforms (TC), and fecal coliforms (FC), viral indicator candidates were explored. Additionally, we also measured physicochemical parameters such as temperature, pH, conductivity, turbidity, chemical oxygen demand (COD_Mn_) and ammonium content (NH_3_-N) in water. We believed this would help reveal the relationship between human enteric viruses and some physicochemical parameters in surface waters. This study aimed to provide beneficial tools to assess the occurrence of enteric viruses in river water.

## Methods

### Sample collection

150 L of water was collected from a depth of 0.5 m below the surface of the sample site proximal to the Jinhe River in Tianjin located at 39°6′58.36”N, 117°13′20.66″E in the center of the city (Additional file 1: Figure S1). The Jinhe River is an important tributary of the Haihe River and is one of the most important rivers in Tianjin, with a full length of 18.5 km and a width of eight meters. It flows through the city center, which contains a large number of commercial, catering, cultural, and entertainment industries on both sides of the river, as well as a considerable residential area.

A total of 48 samples were collected once a month from March 2012 to February 2016 and they were transported in cold storage conditions to the laboratory in approximately 3 h. According to the Chinese Meteorological Administration, the period from March to May was defined as spring, June to August defined as summer, September to November defined as autumn, and December to February defined as winter.

### Quality of surface water samples

All samples were assayed for HPC, TC, and FC on Luria-Bertani agar, M-Endo medium (BD Difco, USA) and M-FC medium (BD Difco, USA) according to the standard membrane filter procedure [17]. Turbidity and conductivity were measured with a HACH 1900C portable turbidity meter (HACH, USA) and a HACH sension5 conductivity meter (HACH, USA), respectively. Chemical oxygen demand (COD_Mn_) and ammonium content (NH_3_-N) were measured according to standard methods [17].

### Virus concentration from water samples using a filter cartridge

Fifty L samples of the river water were filtered through a filter cartridge filled with electropositive granule media (EGM) according to Jin [18]. Then, 3 L of eluent (2% sodium hydroxide, 0.375% glycine, 1.5% sodium chloride, 3% tryptone, 1.5% beef powder) was passed through the column. Eluates were neutralized by the addition of 0.1 mol/L HCl immediately after collection and then 10% PEG was added to the eluates before overnight centrifugation (15,000×g for 30 min at 4 °C). Then, the pellets were resuspended in 40 mL PBS and stored at − 70 °C until further analysis. To evaluate the efficiency of virus recovery, 10^5^ PFU of bacteriophage MS2 was cultivated by confluent lysis on its host strain, *E. coli* (ATCC 15597), and then added to water samples as an indicator and detected using the double layer plaque assay. Virus recovery was calculated using the following eq. (1):

1 $$ \mathrm{Virus}\ \mathrm{recovery}\ \left(\%\right)=\left(B-C\right)/A\times 100\% $$

Where *A* is the number of seeded MS2 into the tested water samples before concentration; *B* is the number of MS2 measured in the final buffered concentrate; and *C* is the number of environmental/background MS2 measured in the final buffered concentrate.

### Viral DNA/RNA extraction

According to the manufacturer’s instructions, viral RNA was extracted from the concentrated viral suspensions using the QIAamp viral RNA mini kit (Qiagen, Hilden, Germany) to detect HRVs, HuNoVs GII, AstVs, and EnVs. UNIQ-10 viral DNA kit (Sangon Biotech) was used for DNA extraction of HAdVs. The purity and concentration of DNA/RNA were determined by a Gene Quant1300 system (GE Healthcare), and samples meeting the purity standards (A260/A280, 1.8–2.0) were used for further analysis. The nucleic acid extraction recovery was evaluated by addition of internal control (IC) RNA to the lysis buffer according to the manufacturer’s instructions (QIAamp Viral RNA Mini Handbook, Qiagen, Hilden, Germany).

### Quantification of viruses by (RT-)qPCR

Reverse transcription was performed using a cDNA first-strand synthesis system (Thermo Fisher Scientific, Waltham, MA) for viruses. 15 μL of template RNA was added to 2 μL of Random Hexamer primer (0.2 μg/μL), incubated for 5 min at 65 °C, and chilled on ice. Then 23 μL of reaction mixture, which contained 8 μL of 5X Reaction Buffer, 2 μL of RevertAid M-MuLV Reverse Transcriptase (200 U/μL), 2 μL RiboLock RNase inhibitor (20 U/μL), 4 μL of 10 mM dNTP Mix, and 7 μL nuclease-free water, was added to the samples. The mixtures were incubated for 5 min at 25 °C, for 60 min at 45 °C, and the reaction was terminated by heating at 70 °C for 5 min in a 2720 thermocycler (Applied Biosystems, USA) to synthesize cDNA. The mixtures were then held at 4 °C for qPCR amplification.

A total volume of 20 μL was used for qPCR, including 2 μL of DNA from HAdVs or cDNA from EnVs, AstVs, HRVs, and HuNoVs GII; 10 μL PCR SuperMix-UDG (Platinum PCR SuperMix-UDG, Invitrogen, USA); 0.5 μL of each primer (10 μmol/L); 0.5 μL of TaqMan probe (5 μmol/L), and; 6.5 μL nuclease-free water. The reaction was performed in an ABI sequence detection system 7300 (Applied Biosystems, USA) under the following conditions: 95 °C for 30 s, followed by 40 cycles of 95 °C for 30 s and 60 °C for 1 min. All qPCR analyses were performed in triplicate with positive controls for each target and DEPC-treated water as the negative controls to ensure cycling efficiencies. All primers and probes (Invitrogen, Shanghai, China) labeled with FAM detector dyes and TAMRA quencher dyes are shown in Additional file 1: Table S1 [18–22].

The standard curves for the quantification of HRVs, HuNoVs GII, AstVs, EnVs, HAdVs, and HCVs (Hepatitis C virus) were obtained by analyzing 10-fold serial dilutions of viral RNA or DNA standards (SI Additional file 1: Figures S2 − S7) [18]. The detailed information pertaining to how viral RNA or DNA standards were prepared as shown in the SI for MM.

### Inhibition control and calculations for virus concentration

To avoid inhibition occurring in the RT-qPCR reaction, 10^5^ genome copies (GCs)/reaction of an HCV RNA IC was added to 2 μL nucleic acids (10- or 50-fold dilution or none) extracted from the samples or DEPC water (blank control) and then assayed using RT-qPCR. If the threshold cycle value (Ct) of the blank control was one cycle less than that of the HCV RNA IC mixed with the nucleic acid extracts from samples, inhibition of the reaction had occurred, and dilutions of the nucleic acid extracts were performed until no inhibition was observed [18]. As background controls, all samples should be verified for a lack of indigenous HCV using RT-qPCR prior to the inhibition check.

The quantification of HCV RNA IC was carried out using real-time procedures following the same conditions as for virus detection with Primer and TaqMan probe sequences listed in Additional file 1: Table S1. The equation for calculating the sample inhibition is:

2 $$ \mathrm{Virus}\ \mathrm{recovery}\ \left(\%\right)=\left(A-B\right)/A\times 100\% $$

Where *A* is the GCs of IC/reaction in the blank control, *B* is the measured GCs of IC/reaction mixed with the nucleic acid extracts in the tested water samples.

### Calculations for virus concentration

HRVs, HuNoVs GII, AstVs, and EnVs concentrations in all water samples were quantified using the following eq. (3) and HAdVs concentration were quantified using the following eq. (4):

3 $$ \mathrm{Virus}\ \left(\mathrm{GCs}\right)=\mathrm{GCs}/ reaction\times 80\ \upmu \mathrm{L}/2\ \upmu \mathrm{L}\times \mathrm{Ve}/140\ \upmu \mathrm{L}\times \mathrm{N} $$

4 $$ \mathrm{Virus}\ \left(\mathrm{GCs}\right)=\mathrm{GCs}/ reaction\times 50\ \upmu \mathrm{L}/2\ \upmu \mathrm{L}\times \mathrm{Ve}/200\upmu \mathrm{L}\times \mathrm{N} $$

Where 2 μL was the volume of sample per reaction tube, and the 140 and 80 μL are the volume of sample extracted and the RNA extract volume of HRVs, HuNoVs GII, AstVs and EnVs, respectively. The 200 and 50 μL are the volumes of sample extracted and the DNA extract volume of HAdVs, respectively. The Ve is the volume of final buffered concentrate (μL). N represents dilution of the nucleic acid extract.

### Statistical analysis

Statistical analyses were performed using SAS9.2 and R language. Viral distributions were compared using the non-parametric Kruskal-Wallis test. Correlations between the virus positive rate and season were analyzed using Fisher’s Exact Test. The run-length testing method was used to analyze the differences in viral concentrations between seasons. Virus concentrations within the same month of different years were analyzed using a Friedman test. Pearson test was calculated using R Studio to measure the strength of associations between enteric virus concentrations and detection indices. This was followed by a Student-Newman-Keuls-q test to analyze the correlation between these variables.

## Results and discussion

### The characteristics of water quality in Jinhe River

Table 1 summarizes the physicochemical and bacterial parameters of water samples from the Jinhe River during the periods from March 2012 to February 2016. It showed that all the parameters fluctuated continuously through the year, exhibiting periodic variation in a seasonal pattern (Additional file 1: Figures S8 and S9). Physicochemical parameters e.g., turbidity, water temperature, COD_Mn_, NH_3_-N, and bacterial parameters e.g., HPC, TC, and FC reached the maximal value during warm months (May–September) while the maximal pH and conductivity occurred in the cold months (November–February).

**Table 1:** Characteristics of bacterial indices and physicochemical parameters of water samples from Jinhe River

Season	Variation range								
	HPC (CFU/ml)	TC (CFU/100 ml)	FC (CFU/100 ml)	T (°C)	pH	Turbidity (NTU)	COD_Mn_ (mg/L)	NH_3_-N (mg/L)	Conductivity (μs/cm)
Spring	4.0 × 10^2^ −1.56 × 10^4^	7.5 × 10^2^ − 1.6 × 10^4^	1.0 × 10^2^ -3.5 × 10^3^	8.7–21	6.00–6.70	2.42–15.77	4.54–14.61	0.54–5.43	550–643
Summer	3.5 × 10^3^ −2.8 × 10^5^	1.0 × 10^2^ −5.5 × 10^4^	2.7 × 10^2^ -3.8 × 10^4^	26.5–32	6.00–6.90	6.55–29.23	8.93–22.40	0.80–9.54	514–647
Autumn	6.5 × 10^2^ −4.5 × 10^5^	2.5 × 10^2^ -1.1 × 10^5^	1.8 × 10^3^ -6.7 × 10^4^	6.3–27	6.00–7.40	4.07–24.23	9.53–22.30	1.54–7.53	605–828
Winter	5.5 × 10^1^ −2.9 × 10^3^	5.0 × 10^2^ -1.7 × 10^4^	2 × 10^4^ -2.0 × 10^4^	2–6.4	6.40–7.40	2.63–7.93	2.50–10.98	0.54–3.56	624–868
PA DEP reference values		5.0 × 10^3^	2.0 × 10^2^						

Abbreviations: *HPC* heterotrophic plate counts, *TC* total coliforms, *FC* fecal coliform, *T* water temperature, *NTU* nephelometric turbidity unit, *COD*_*Mn*_ chemical oxygen demand, *NH*_*3*_*-N* ammonium content

Fecal indicator TC and FC were detected in all samples collected across the four-year survey and 23 of 48 (47.9%) samples exceeded the regulations provided by the Pennsylvania Department of Environmental Protection (PA DEP) for TC (5000 CFU/100 mL) [23] at concentrations ranging from 5.3 × 10^3^ to 1.1 × 10^5^ CFU/100 mL, which were observed mainly between May and September. Concentrations of the FC in all samples ranged between 2.7 × 10^2^ to 6.7 × 10^4^ CFU/100 mL in May–September, which exceeded the PA DEP regulations for fecal coliforms (200 CFU/100 mL) during the season (May 1 through September 30) [23]. For this reason, high concentrations of TC and FC are likely indicative of high levels of human fecal contamination in warm months.

### Detection of enteric viruses in Jinhe River

**Fig. 1:**
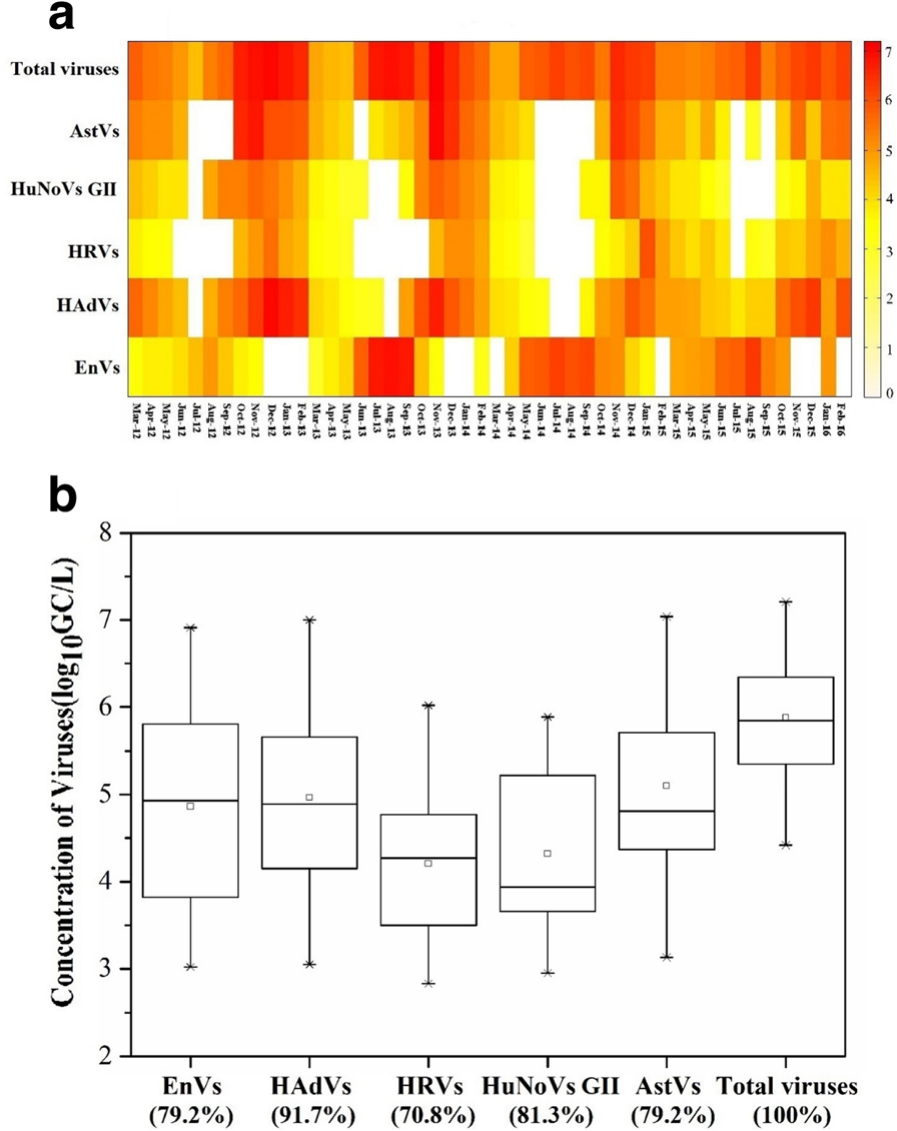
**a** Heat map of enteric viruses detected in water samples from the Jinhe River between March 2012 and February 2016 (*n* = 3). Different shades represent the different virus concentrations (log_10_GC/L); (**b**) Comparison of virus concentrations in virus-positive water samples detected by RT-qPCR (*n* = 144). The median value is represented by a line inside the box, geometric mean (o), 95% confidence intervals (bars). The percentage of occurrence is given in parenthesis

Figure 1a illustrated the occurrence and the abundances of enteric viruses in water samples from the Jinhe River between March 2012 and February 2016. It showed that all types of enteric viruses can be found in the Jinhe River and at least one of the target viruses could be detected positively every month. In particular, all targeted viruses were positive simultaneously in 22 of 48 (45.8%) samples, which were mainly in the periods between March–May and October–November. Among all the observed viruses (Fig. 1b), HAdVs were the most prevalent with a detection frequency of 91.7% (44/48). Its geometric mean concentration in positive samples was 4.96 log_10_GC/L. For HuNoVs GII, the mean concentration of 4.32 log_10_ GC/L was the next most common with a detection frequency of 81.3% (39/48). AstVs and EnVs were detected in 79.2% (38/48) of samples with the highest mean concentrations of 5.10 log_10_GC/L and 4.86 log_10_ GC/L, respectively. HRVs were the least prevalent virus and were detected in 34 of 48 (70.8%) samples. They also had the lowest viral level of 4.21 log_10_GC/L. There was a significant difference in the average concentrations of various viruses (Kruskal-Wallis, *P* < 0.05).

Generally, high concentrations of enteric viruses are found in the feces of infected patients [24]. Due to the their resistance to unfavorable circumstances and wastewater treatment, enteric viruses can persist in the wastewater treatment process (WWTP) effluent and retain survivability in the environment in recipient rivers for long periods [25]. Considering the Jinhe River as a river, which spans across Tianjin city, the frequent presence of these enteric viruses in water, even at low concentrations, may pose a public health threat to those in surrounding residences.

Figure 1a also exhibited the total load of observed enteric viruses in the Jinhe River, fluctuating from 4.42 to 7.20 log_10_ GC/L with the maximal value around November annually and minimally around May of the following year. There were no significant differences in the total viral loads between the same months during the four-year sampling period (Friedman test, *P* > 0.05). During the four-year sampling period, individual enteric viruses in water samples also exhibited seasonal patterns in their occurrence and concentrations, similar to the physicochemical and microbial parameters of water samples (Table 2, Run-length testing method, P < 0.05). Significant correlations were found between viral occurrence and season types for all five target viruses (Fisher’s Exact Test, *P* < 0.01). For acute gastroenteritis viruses, including HAdVs, HRVs, HuNoVs GII and AstVs, their concentrations in the river were higher during cold weather rather than the warm weather months. They were positively detected every month in the seasons of spring and winter at concentrations ranging from 3.39 to 7.00, 2.83 to 6.02, 3.01 to 5.56, and 4.01 to 6.47 log_10_ GC/L for HAdVs, HRVs, HuNoVs GII, and AstVs, respectively. Only 16.7% of HRVs and 33.3% of HuNoVs were detected in summer at concentrations ranging from 0 to 3.29 and 0 to 2.95 log_10_ GC/L, respectively. In contrast, EnVs concentrations in the river were higher during the warm weather rather than the cold weather months. A detection rate of 100% every month in summer at concentrations ranging from 4.04 to 6.91 log_10_ GC/L but was only 33.3% in winter at concentrations ranging from 0 to 4.06 log_10_ GC/L.

**Table 2:** Occurrence of enteric viruses in seasons (*n* = 36)

Season^a^	Detection rate (%)				
	EnVs	HAdVs	HRVs	HuNoVs GII	AstVs
Spring	91.7 ± 16.7	100 ± 0	100 ± 0	100 ± 0	100 ± 0
Summer	100 ± 0	66.7 ± 27.2	16.7 ± 33.3	33.3 ± 27.2	41.7 ± 31.9
Autumn	91.7 ± 16.7	100 ± 0	66.7 ± 27.2	91.7 ± 16.7	75 ± 16.7
Winter	33.3 ± 27.2	100 ± 0	100 ± 0	100 ± 0	100 ± 0

^a^Defined according to the Chinese Meteorological Institute: spring, from March to May; summer, from June to August; autumn, from September to November; winter, from December to February

Although high concentrations of acute gastroenteritis viruses were identified in cold weather (November–February), this may suggest that the river has high concentrations of human fecal contamination but concentrations of TC at the sampling site were generally low (maximum of 5.4 × 10^3^ CFU/100 mL). This phenomenon may be a result of the high incidence of acute infectious gastroenteritis or diarrhea cases in clinics over the same timeframe. High infection rates of HRVs, HuNoVs GII, and AstVs among children in the autumn and winter have been observed in Tianjin, the river basin of the sampling site [26]. Also, a high incidence of diarrhea associated with EnVs occurred in the summer in China [27]. Therefore, all of these results suggest that enteric viruses in the river are strongly associated with the clinical epidemiology of the river catchment area. Correspondingly, TC could not predict the total enteric virus presence but was more specific to acute gastroenteritis viruses.

### Correlation between virus concentrations and physicochemical parameters in the Jinhe River

**Fig. 2:**
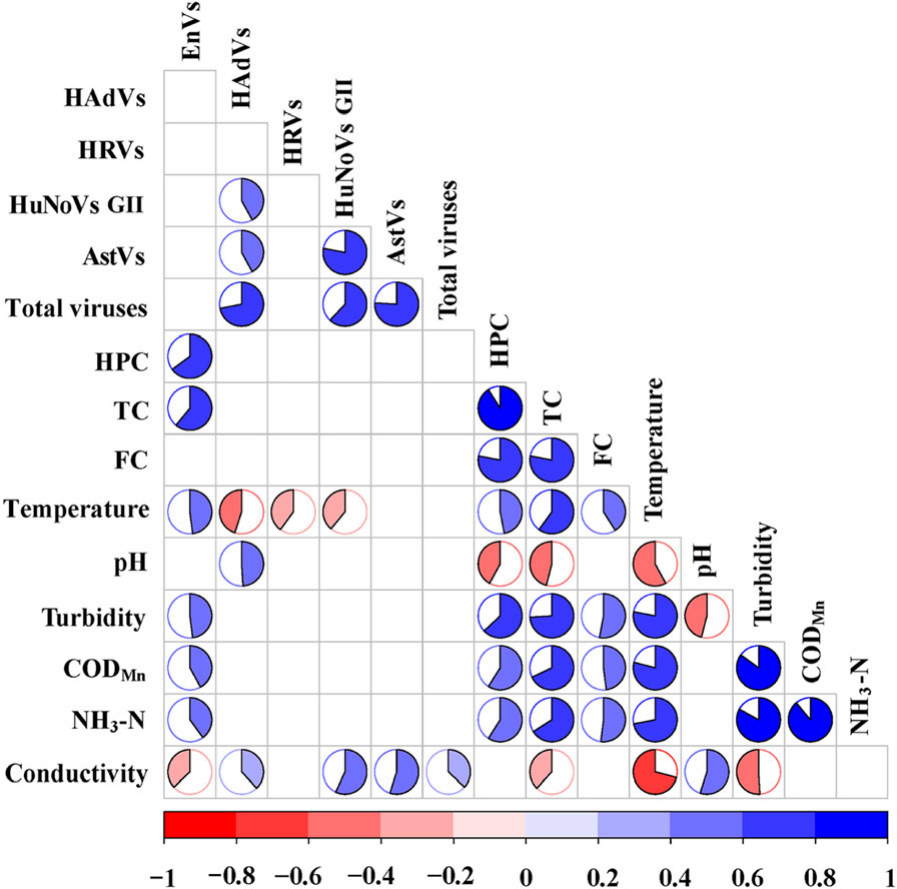
Correlation matrix between virus concentration and water parameters analyzed by Pearson correlation analysis (*p* < 0.01). The range of the coefficients is from − 1 to 1, where − 1 indicates a direct negative correlation, 0 indicates no correlation, and 1 indicates a direct positive correlation

Enteric viruses were significantly correlated with all of the measured physicochemical indices (Fig. 2). EnVs showed the strongest positive correlation with temperature, turbidity, COD_Mn_, and NH_3_-N while they were negatively correlated with conductivity. In contrast, HAdVs, HRVs, AstVs, and HuNoVs GII were significantly correlated with temperature (negatively) or conductivity (positively). Above all, conductivity was the only physicochemical index that was positively correlated with total viral concentration (*P* < 0.05, Kendall’s Tau-b).

Among all the physicochemical parameters, enteric viruses had the strongest correlation with water temperature with the exception of AstVs (Fig. 2 and Additional file 1: Figure S10; Pearson, *P* < 0.01). For acute gastroenteritis viruses, there was a significant negative correlation between their concentration and temperature. Viral concentrations remained the highest when temperatures ranged from 2 °C to 6 °C, while viral concentrations declined to the lowest when temperature was between 27 °C and 32 °C. This is likely due to their high clinical prevalence in winter as well as the lower temperatures, as higher viral persistence has been reported in vitro at lower temperatures [28, 29]. In contrast, EnVs were positively correlated with temperature, and EnVs concentrations increased to the highest value when temperature exceeded 16 °C. There were significant differences between viral concentrations under different temperature conditions (Student-Newman-Keuls-q test), which was consistent with the differences in viral concentrations under different seasonal conditions.

### Correlation between virus concentrations and bacterial indices in the Jinhe River

**Fig. 3:**
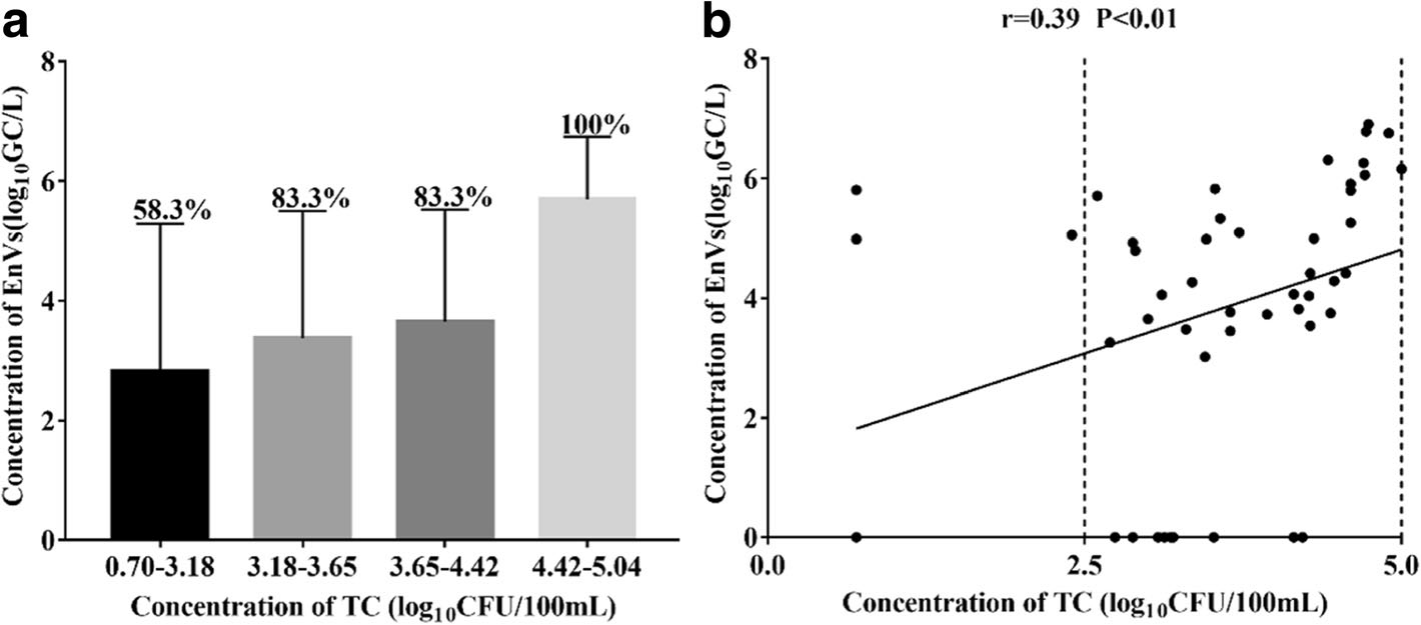
Relationships between EnVs and total coliforms, which were selected from the quartiles of ranked observations. The percentage of EnVs occurrence is given in (**a**), the linearity between EnVs and total coliforms is shown in (**b**)

Through Pearson analysis, only EnVs concentration in the Jinhe River showed a significant positive correlation with TC (Fig. 2; Pearson, P < 0.01). Meanwhile, only 58.3% (7/12) of the samples with TC levels that were lower than 3.18 log_10_CFU/100/mL was positive for EnVs but for the samples whose TC levels exceeded 4.42log_10_CFU/100 mL, we found that EnVs were significantly positive and there was a significant increase observed in the EnVs concentration (Fig. 3, Student-Newman-Keuls-q test). These data indicate that EnVs were more likely to be detected when the concentration of TC increased (Fig. 3, χ^2^ test; *P* < 0.05). Therefore, TC may be a potentially promising candidate to assess the degree of EnVs contamination in the surface water across the city due to its stability and ability to identify origin of enteric viruses. This is true even if the results of several studies [30, 31] on drinking water demonstrated that TC did not reflect the occurrence of EnVs due to the frequent occurrence of EnVs in water which met current bacteriological standards. In effect, EnVs may be suggested as an alternative viral indicator of fecal pollution.

Significant positive correlations between the concentrations of HuNoVs GII, HAdVs, and AstVs can be observed. Furthermore, there were significant decreases in acute gastroenteritis virus concentrations when HPC exceeded 6.07 log_10_CFU/100 mL (Additional file 1: Figure S10, Student-Newman-Keuls-q test) and also in HRVs concentration when the TC exceeded 4.42 log_10_CFU/100 mL. However, no significant correlation was observed between concentrations of acute gastroenteritis viruses with EnVs, TC, HPC, or FC (Fig. 2; Pearson, *P* < 0.01), indicating failure to predict the presence of non-EnVs enteric viruses in surface water using bacterial indices. It also meant acute gastroenteritis viruses should not be an alternative indicator of fecal or EnVs pollution even if there is prolonged virus persistence in the environment. Some previous studies [32, 33] have also suggested that bacteriological indicators e.g., TC concentrations do not accurately reflect human viral (HRVs, HuNoVs GII, and HAdVs) dispersal in marine waters, individual groundwater, and contamination of shellfish by sewage-derived viral pathogens.

## Conclusions

A high occurrence of enteric viruses in a specific seasonal pattern were observed in the Jinhe River over a four-year survey. TC were significantly positively correlated with EnVs concentrations while bacterial indices and acute gastroenteritis viruses did not show any consistent significant correlations. These data indicate that TC seems to be a potentially promising candidate to assess the degree of EnVs contamination but fails to predict the presence of HAdVs, HRVs, AstVs, and HuNoVs GII in the surface water across the city of Tianjin. Furthermore, any one of HAdVs, HRVs, AstVs, and HuNoVs GII cannot be used as an alternative indicator of fecal or EnVs pollution in the environment.

## Additional file

Additional file 1: **Figure S1.** contains the map of the water sampling site. **Figures S2-S7.** contain the standard curves for the quantification of HRVs, HuNoVs GII, AstVs, EnVs, HAdVs and HCVs. **Figures S8-S9.** contain the comparison of physicochemical and bacterial indices in water samples. **Figure S10.** contains the virus concentrations in water samples compared with season, temperature and bacterial indexes. **Table S1.** contains the primers and probes used in this study. (DOCX 1736 kb)

## Abbreviations

AstVs: Astroviruses
COD_Mn_: Chemical oxygen demand
EnVs: Enteroviruses
FC: Fecal coliform
HAdVs: Adenoviruses
HCVs: Hepatitis C virus
HPC: Heterotrophic plate counts
HRVs: Rotaviruses
HuNoVs GΙΙ: Noroviruses GΙΙ
NH_3_-N: Ammonium content
T: Water temperature
TC: Total coliforms
