# Supplementary material for: Total coliforms as an indicator of human enterovirus presence in surface water across Tianjin city, China
Source: BMC Infect Dis. 2018 Nov 1;18:542. doi: 10.1186/s12879-018-3438-5 (PMC6211496; doi:10.1186/s12879-018-3438-5)
Supplement: Supplementary file 1 — Figure S1. contains the map of the water sampling site. Figures S2-S7. contain the standard curves for the quantification of HRVs, HuNoVs GII, AstVs, EnVs, HAdVs and HCVs. Figures S8-S9. contain the comparison of physicochemical and bacterial indices in water samples. Figure S10. contains the virus concentrations in water samples compared with season, temperature and bacterial indexes. Table S1. contains the primers and probes used in this study. (DOCX 1736 kb) [file 12879_2018_3438_MOESM1_ESM.docx]

Supporting information

Title:

Total coliforms as an indicator of human enterovirus presence in surface water across Tianjin city, China

**Author names and affiliations:**

Jing Miao^1^, Xuan Guo^1,2^, Weili Liu^1^, Dong Yang^1^, Zhiqiang Shen^1^, Zhigang Qiu^1^, Xiang Chen^1^, Kunming Zhang^1^, Hui Hu^1^, Junwen Li^1,*^ and Min Jin^1,*^

^1^ Tianjin Institute of Environmental ＆Operational Medicine, Key Laboratory of Risk Assessment and Control for Environment & Food Safety, Tianjin 300050, China

^2^ Research Institution of Chemical Defense, Beijing 102205, China

^*^ Correspondence: [jinminzh@126.com(Min](mailto:jinminzh@126.com(Min) Jin); [junwen9999@hotmail.com(Junwen](mailto:junwen9999@hotmail.com(Junwen) Li)

**Figure legends**

**Figure S1 Map of the water sampling site.**

**Figure S2 Standard curve (HRV).** Fluorescent amplification curves of RT-qPCR for RNA standards of HRV with concentrations of 1.18×10^7^ copies/μl ~ 1.18 copies/μl from left to right (a), and the corresponding standard curve for HRV RNA (Y=-3.359X+40.050). The linear regression coefﬁcient (r^2^) is 0.999 (b). Water was used as a non-template control.

**Figure S3 Standard curve (HuNoV GII).** Fluorescent amplification curves of RT-qPCR for RNA standards of HuNoV GII with concentrations of 1.2×10^7^ copies/μl ~ 12 copies/μl from left to right (a), and the corresponding standard curve for HuNoV GII RNA (Y=-3.180X+39.245). The linear regression coefﬁcient (r^2^) is 0.991 (b). Water was used as a non-template control.

**Figure S4 Standard curve (EnV).** Fluorescent amplification curves of RT-qPCR for RNA standards of EnV with concentrations of 5.88×10^7^ copies/μl ~5.88copies/μl from left to right (a), and the corresponding standard curve for EnVs RNA (Y =-3.002X+37.989). The linear regression coefﬁcient (r^2^) is 0.998 (b). Water was used as a non-template control (gray lines).

**Figure S5 Standard curve (AstV).** Fluorescent amplification curves of RT-qPCR for RNA standards of AstV with concentrations of 1.83×10^7^ copies/μl ~1.83copies/μl from left to right (a), and the corresponding standard curve for AstV RNA (Y =-3.571X+42.941). The linear regression coefﬁcient (r^2^) is 0.990 (b). Water was used as a non-template control (gray lines).

**Figure S6 Standard curve (HAdV).** Fluorescent amplification curves of qPCR for plasmid standards of HAdV with concentrations of 4.3×10^7^ copies/μl ~ 4.3 copies/μl from left to right (a), and the corresponding standard curve for HAdV (Y=-3.566X+44.258). The linear regression coefﬁcient (r^2^) is 0.995 (b). Water was used as a non-template control.

**Figure S7 Standard curve (HCV).** Fluorescent amplification curves of qPCR for plasmid standards of HCV with concentrations of 2.3×10^7^ copies/μl ~ 2.3 copies/μl from left to right (a), and the corresponding standard curve for HCV (Y=-3.263X+47.251). The linear regression coefﬁcient (r^2^) is 0.990 (b). Water was used as a non-template control.

**Figure S8** Comparison of physicochemical indices in water samples from Jinhe River, measured from March 10, 2012 to February 10, 2016. Temperature measured in °C, Turbidity in NTU, Conductivity in µs/cm, Chemical oxygen demand and Total ammonium content both in mg/L.

**Figure S9** Comparison of bacterial indices in water samples from Jinhe River, measured from March 10, 2012 to February 10, 2016.

**Figure S10** (a) Seasonal distribution of viruses in water samples from the Jinhe River (n=36); (b) Virus concentrations (log_10_GC/L) in water samples from the Jinhe River compared with temperature (℃), which were selected from the quartiles of ranked observations; (c) Virus concentrations (log_10_GC/L) in water samples from the Jinhe River compared with bacterial indexes (log_10_CFU/mL), which were selected from the quartiles of ranked observations.

**Table S1** Primers and probes used in this study.

**
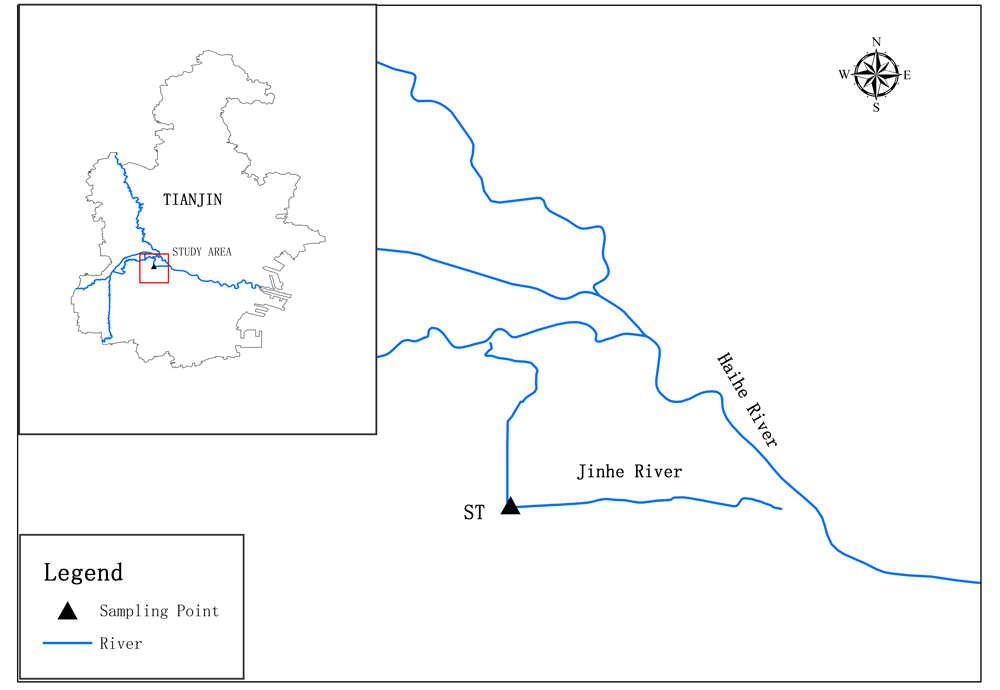
**

**Figure S1**


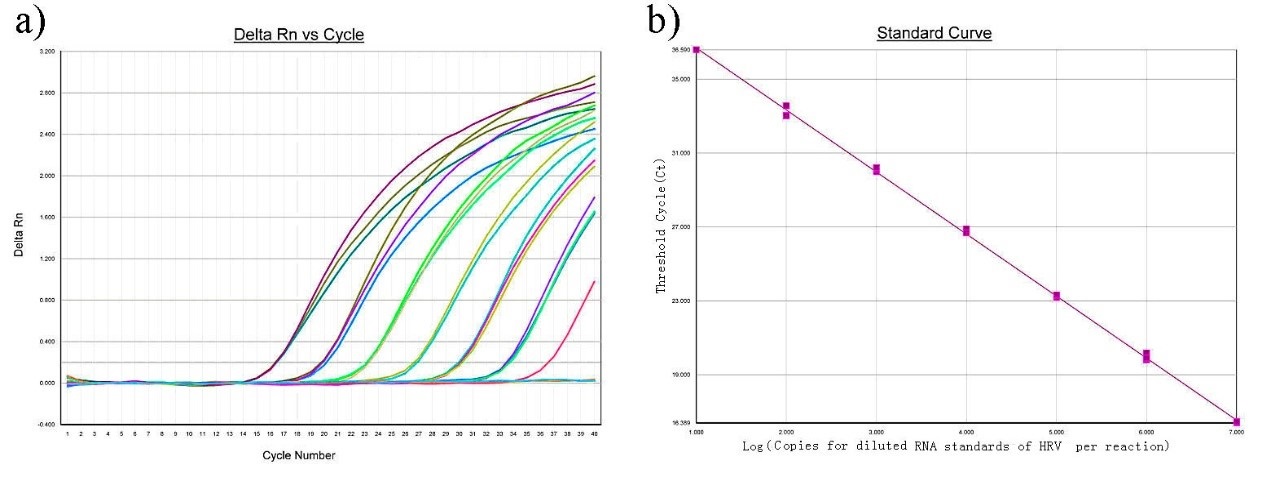


**Figure S2**


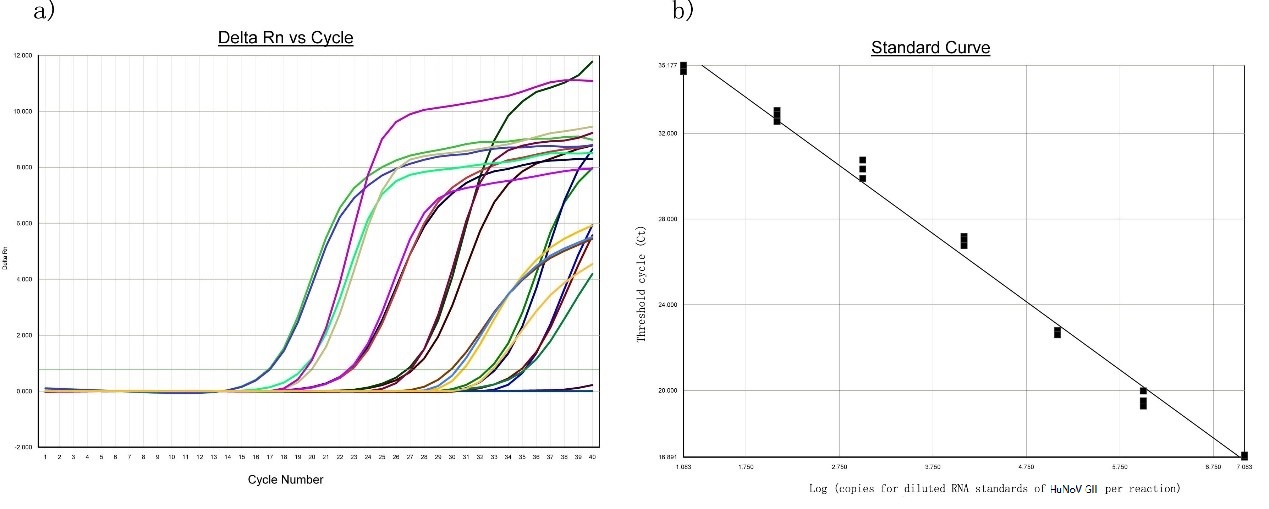


**Figure S3**


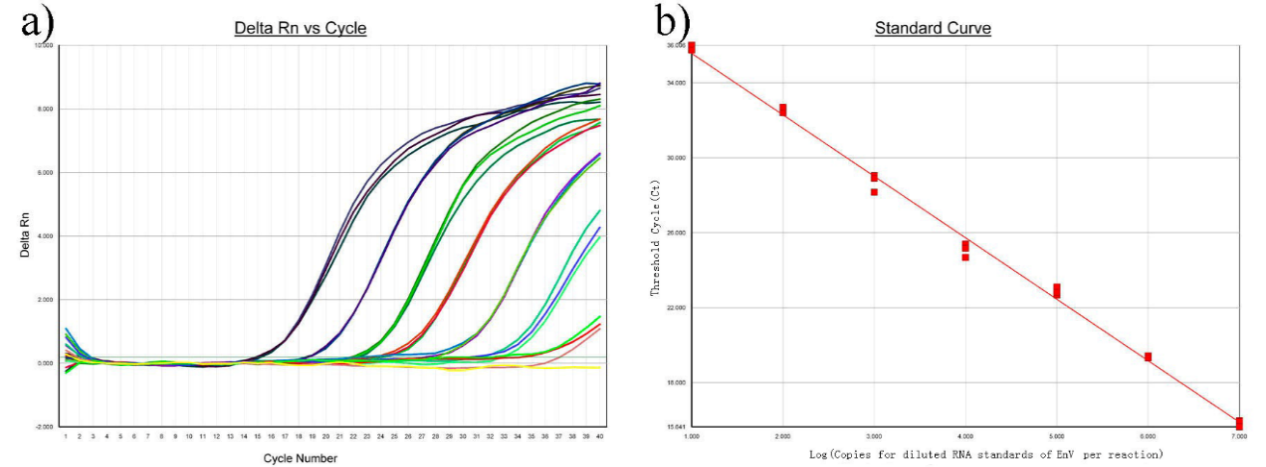


**Figure S4**
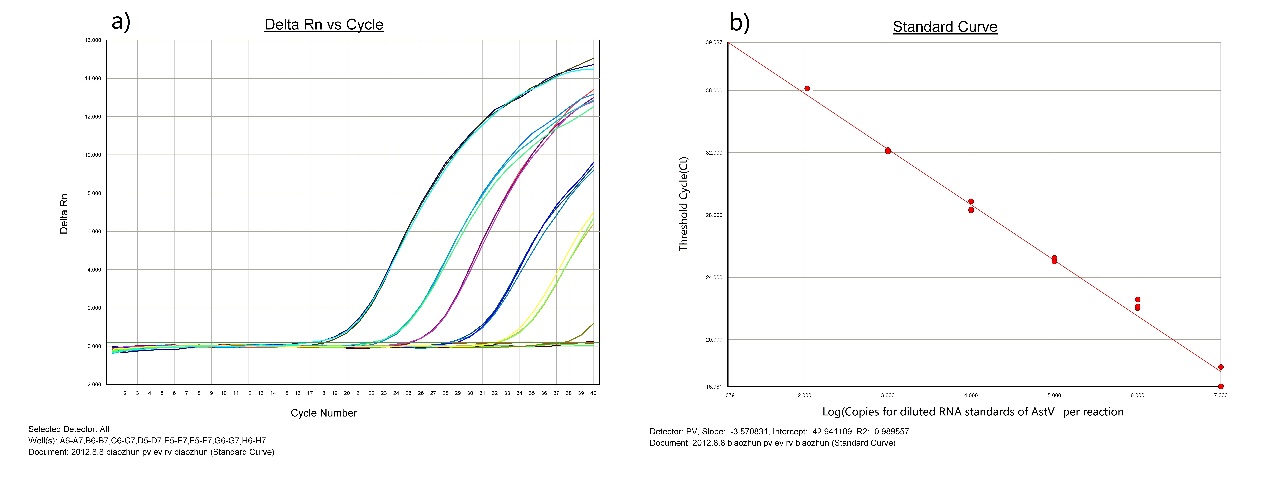


**Figure S5**


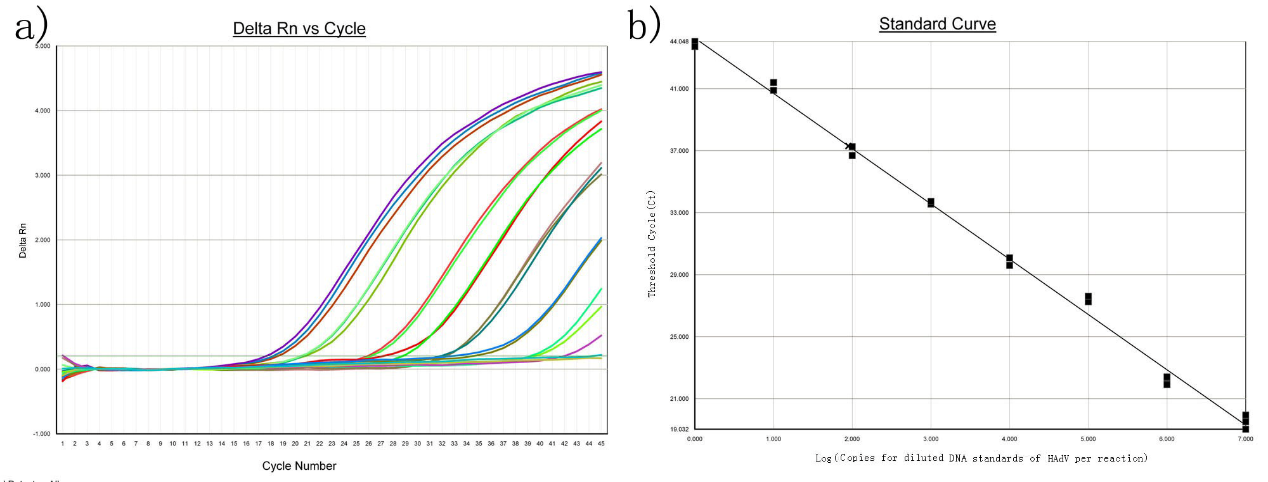


**Figure S6**


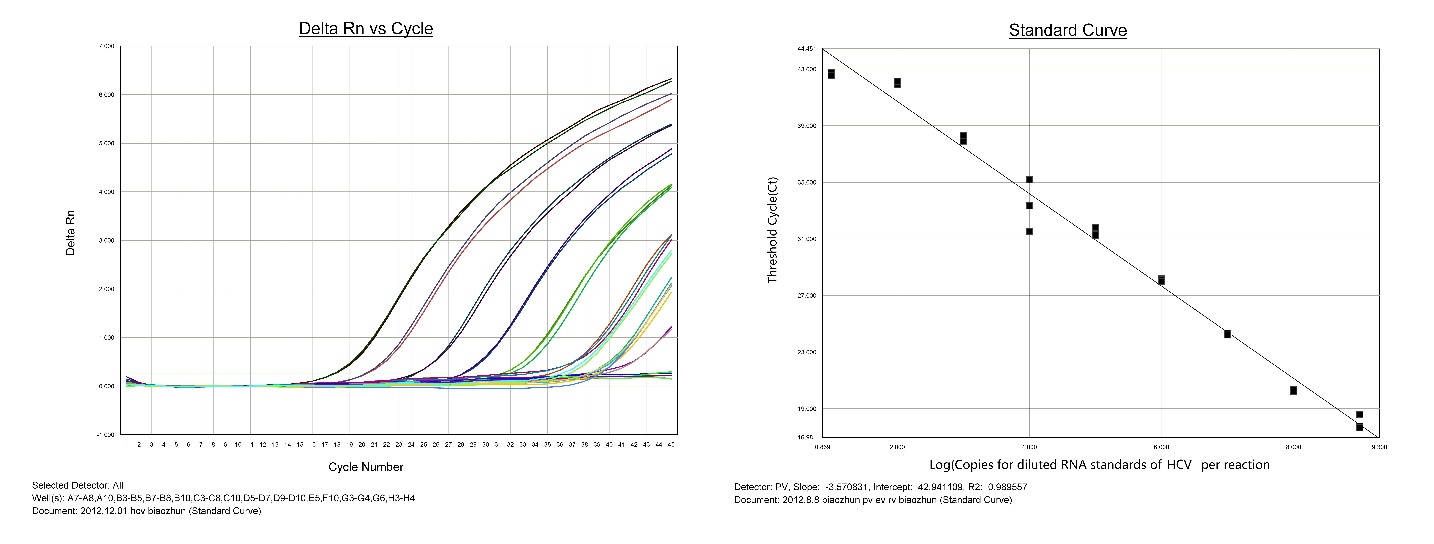


**Figure S7**


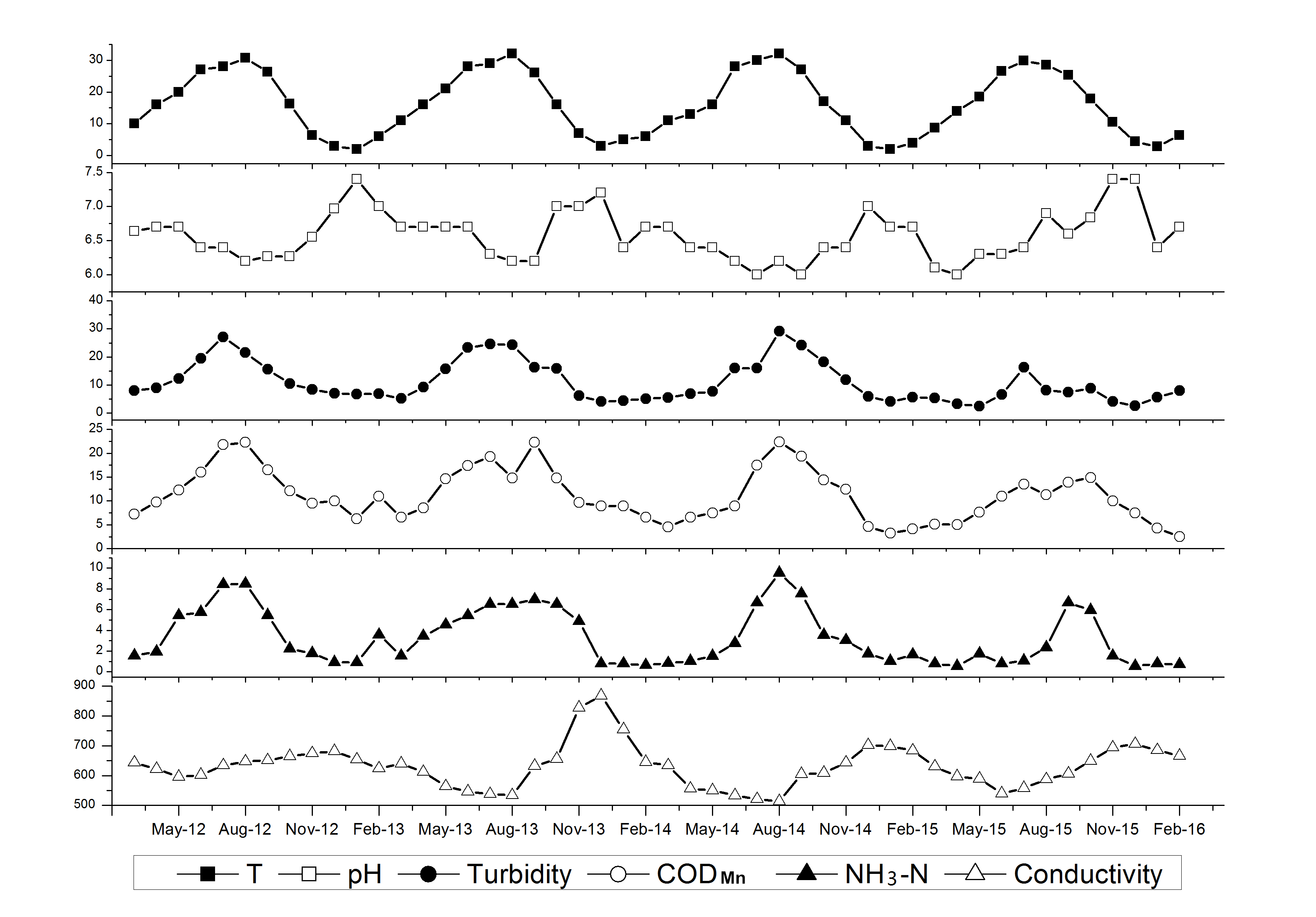


**Figure S8**


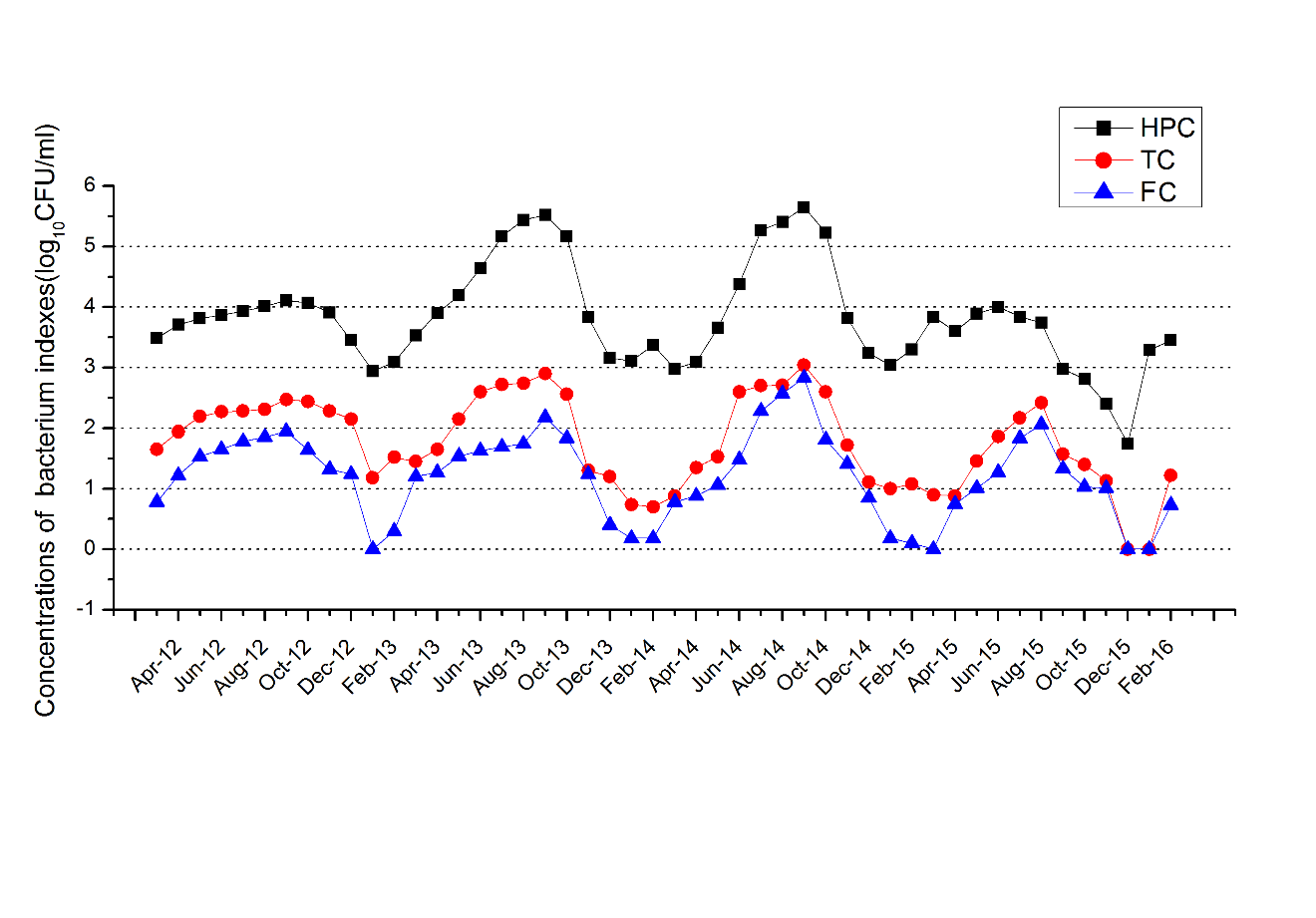
**Figure S9**


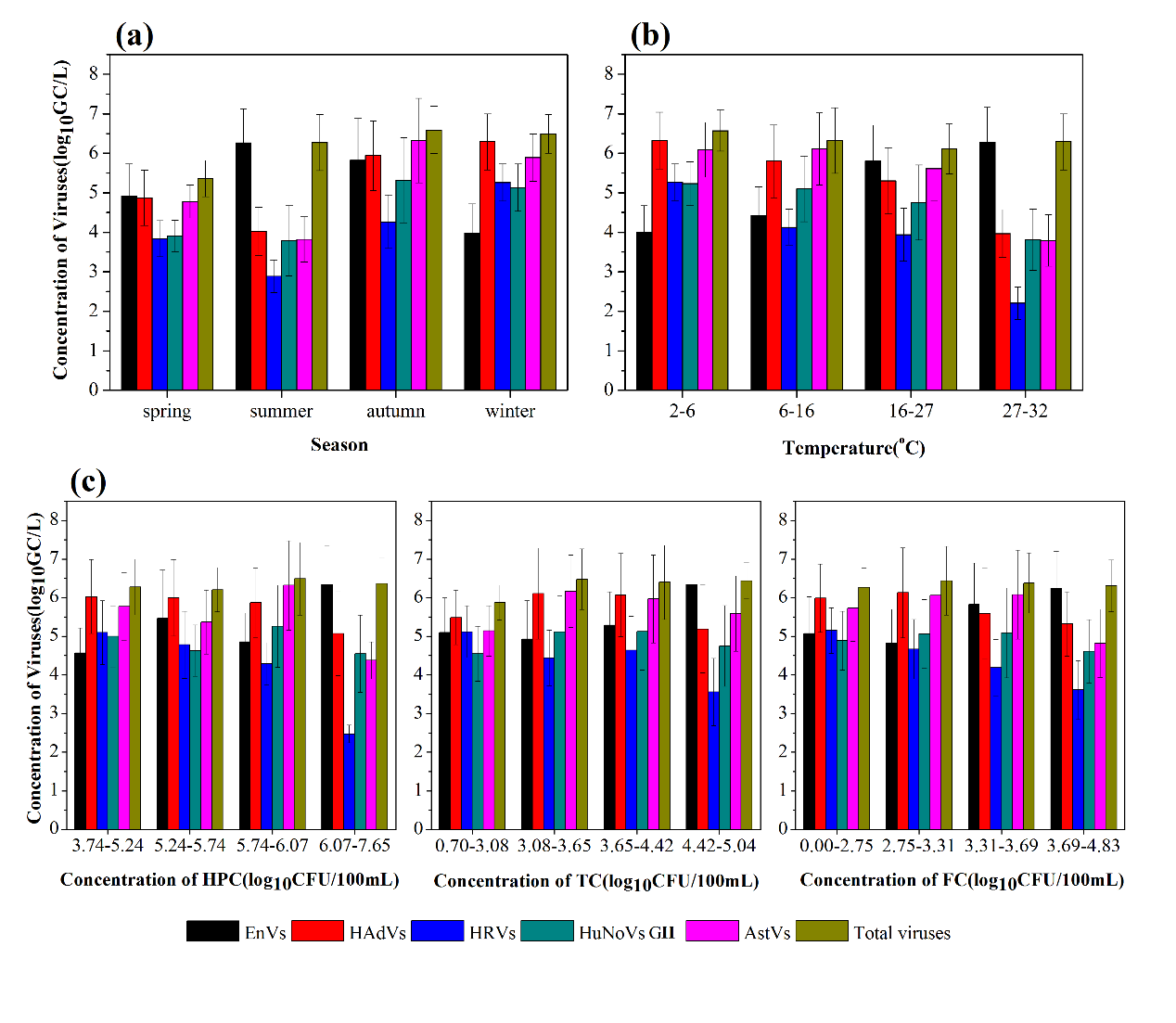


**Figure S10**

**Table S1**

| **Targets** | **Primers or probes** | **Sequences( 5′–3′)** | **Product**  **length (bp)** | **Reference** |
| --- | --- | --- | --- | --- |
| HRVs | RV-Pf | TGTCTGTATTATCCAACTGAAGCAAGT | 86 | [18] |
|  | RV-Pr | CCCTTTGTAAGAAACATTTGCGA |  |  |
|  | RV-TaqMan | HEX-TCAAATCAATGATGGTGACTGGAAAGACT-BHQ1 |  |  |
| AstVs | AST-Q-F | CCGAGTAGGATCGAGGGT | 90 | [21] |
|  | AST-Q-R | GCTTCTGATTAAATCAATTTTAA |  |  |
|  | AST- TaqMan | FAM-CTTTTCTGTCTCTGTTTAGATTATTTTAATCACC-TAMRA |  |  |
| EnVs | EV-U-Pf | GTGGCRGTGGCTGCGYT | 204 | this study |
|  | EV-U-Pr | ACCCAAAGTAGTCGGTTCCGC |  |  |
|  | EV-U-TaqMan | FAM-ATTAGCCGCATTCAGGGGCCGGA-TAMRA |  |  |
| HAdVs | EAdV-Pf | AACTTTCTCTCTTAATAGACGCCCC | 87 | [20] |
|  | EAdV-Pr | TGTCCACTAGTCCAAGAGGTGC |  |  |
|  | EAdV-TaqMan | FAM-GCTGACACGGGCACTCTTCGC-TAMRA |  |  |
| HuNoVs | COG-II-F | CARGARBCNATGTTYAGRTGGATGAG | 98 | [19] |
|  | COG-II-R | TCGACGCCATCTTCATTCACA |  |  |
|  | RING2- TaqMan | FAM-TGGGAGGGCGATCGCAATCT-TAMRA |  |  |
| HCVs | CV-F2 | ATGGCGTTAGTATGAGTGTCGT | 223 | [22] |
|  | CV-R2 | CAAGCACCCTATCAGGCAGT |  |  |
|  | CV- TaqMan | FAM-CCATAGTGGTCTGCGGAACCGGT/139-161-TAMRA |  |  |
